# Recognition of Diffuse Pustules and Hemorrhagic Crusts as Early Warning Signs of Invasive Candidiasis in a Patient With Toxic Epidermal Necrolysis

**DOI:** 10.1002/ccr3.71451

**Published:** 2025-11-16

**Authors:** Saman Al‐Zahawi, Fatemeh Saberi, Tahereh Soori, Elham Yousefi, Kamran Balighi

**Affiliations:** ^1^ Department of Dermatology Razi Hospital, Tehran University of Medical Sciences (TUMS) Tehran Iran; ^2^ Department of Infectious Diseases Razi Hospital, Tehran University of Medical Sciences (TUMS) Tehran Iran; ^3^ Autoimmune Bullous Diseases Research Center Razi Hospital, Tehran University of Medical Sciences Tehran Iran

**Keywords:** dexamethasone, IgA nephropathy, IVIG, oral fluconazole, systemic candidiasis, TEN, toxic epidermal necrolysis

## Abstract

Toxic Epidermal Necrolysis (TEN) is an acute, uncommon, extensive, and life‐threatening dermatological condition, which is caused by a delayed adverse response of the skin to a particular type of drug. Herein, we report a 41‐year‐old female known case of IgA nephropathy admitted for having TEN after a recent intake of Deflazacort, developed systemic candidiasis during admission, and was successfully treated with oral fluconazole. Early detection of diffuse pustular and hemorrhagic crusted lesions with immediate antifungal treatment in TEN is crucial to prevent systemic complications and enhance patient outcomes.

## Graphical abstract

Hemorrhagic crusts of the upper limb, face and neck, in addition to multiple satellite pustular lesions of the leg.

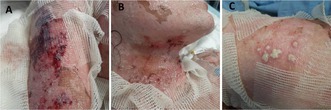

**Summary**

- Early diagnosis of invasive candidiasis—a serious potential complication of TEN—can be guided by the appearance of diffuse pustules and hemorrhagic crusts days before a positive laboratory result

## 1 Introduction

Toxic Epidermal Necrolysis (TEN) is an acute, uncommon, extensive, and life‐threatening dermatological condition, which is caused by a delayed adverse response of the skin to drugs [1]. TEN can occur due to over 100 various medications, usually after 7–21 days from drug intake. Deflazacort was added to the list after a recent report of TEN in association with deflazacort in patients with IgA nephropathy [2]. The early clinical manifestation may be confused with morbilliform skin eruption, which is a commonly seen drug‐related skin eruption. However, the progression of the morbilliform skin eruption into dusky discoloration, involvement of the mucous membrane, and general fatigability should raise suspicion for TEN, before the development of the full picture of the disease in the form of skin detachment and extensive exfoliation. Early discontinuation of the offending drug, early diagnosis, and early institution of treatment may decrease the high mortality rate, which might reach 15%–30% [3]. The high mortality rate is attributed to sepsis by bacterial infection, most commonly *Staphylococcus aureus* and *Pseudomonas aeruginosa* [4]. To decrease major complications in patients with TEN, such as sepsis, electrolyte imbalance, and hypovolemia, patients should be kept in a highly sterilized unit such as an intensive care unit or a burn unit. There are no uniform international guidelines to treat TEN, but Tumor Necrosis Factor α (TNFα) inhibitors, Cyclosporine, IVIG, and systemic corticosteroids have proved helpful drugs in treating TEN [5]. While bacterial sepsis is considered the main cause of death in TEN, opportunistic infections like systemic candidiasis may increase hospitalization and mortality risk [6]. Herein, we report a 41‐year‐old female patient admitted for TEN, who developed systemic candidiasis during admission and was successfully treated with oral fluconazole, which is also a culprit drug in the development of TEN [7].

## 2 Case History/Examination

A 41‐year‐old female with a known case of IgA nephropathy presents to our center with widespread, severe erosive skin and mucous membrane lesions. Following Oxford/AstraZeneca COVID‐19 vaccination, the patient developed IgA nephropathy and was treated with losartan, atorvastatin, prednisolone, and deflazacort. The initial skin lesions started 2 weeks before hospitalization in our center, with widespread erythema and morbilliform skin eruptions just 11 days after she received deflazacort 30 mg/day. The morbilliform skin eruption progressed into dusky discoloration and eventual widespread skin and mucous membrane erosions. She was admitted to our center for having widespread skin and mucous membrane detachment involving more than 80% of the total body surface area (TBSA). The offending medications were discontinued later, 1 week after the skin eruption.

On exam, widespread skin erosion involving more than 80% of the body with positive Nikolsky signs was observed. Also, there were severe oral erosions, which rendered the patient unable to eat or drink, in addition to genital and conjunctival involvement.

### 2.1 Investigations, Diagnosis, and Treatment

She was admitted to the intensive care unit with the diagnosis of TEN. The TEN was attributed to deflazacort based on the tight temporal relationship (rash appearing days after initiation) and the clinical pattern (lack of new lesions after stopping the drug). Mycoplasma‐induced rash and mucositis and lupus‐associated TEN (SLE) were ruled out due to the extensive skin involvement, the patient's overall toxic state, and the absence of other classic SLE features.

Vital signs, laboratory tests, and the SCORTEN score were recorded on the first day and in subsequent days to assess the progression of the disease (Tables 1 and 2). She received intravenous dexamethasone for 3 days before her admission to our center. Dexamethasone injection was continued in our center with prophylactic heparin, topical eye ointments, topical oral medications, intravenous fluid infusion, high‐calorie formula diet, and analgesics and antipyretics. Because of the delayed discontinuation of the offending drug and widespread skin involvement, a new combination treatment of dexamethasone, infliximab, and IVIG was planned for the upcoming days with a close follow‐up of derangement in the lab panel. Treatment with dexamethasone for 1 week did not stop the expansion of the skin erosion. A single dose of infliximab/300 mg was added to her regimen on the third day of her admission. Lastly, because of poor general condition and widespread skin and mucous membrane erosions, IVIG was added on the fourth day with 400 mg/kg for four consecutive days. She was event‐free during the period she received IVIG and for the next 5 days, except for a positive wound culture of *Escherichia coli* , for which systemic antibiotics (colistin, meropenem) were started by the infectious specialist in our center, taking into consideration the drugs that may worsen the already drug‐induced TEN.

**TABLE 1:** Vital signs and SCORTEN score for the first 5 days in the ICU.

Items	Day 1 (admission)	Day 2	Day 3	Day 4	Day 5
SCORTEN score	4	4	4	4	3
Tem	38.5	38.3	38.5	38.2	38.4
BP	130/70	120/70	123/75	130/80	105/60
RR	23	25	21	20	20
PR	105	122	125	120	100
O_2_ saturation	97%	96%	97%	97%	96%
Nikolisky sign	Positive	Positive	Positive	Positive	Positive

**TABLE 2:** Lab test result throughout the three‐week period admission.

Lab test	First week	Second week	Third week	Fourth week
Bleeding profile	Normal	Normal	Normal	Discharged
Ca	5.5	7.9	8.5	
Urine albumin	Proteinuria	Proteinuria	Proteinuria	Proteinuria
CBC	H.b 11	H.b 10	H.b 11	Discharged with nearly normal CBC
	Wbc 15,000	Wbc 19,000	Wbc 13,000	
	Plt 200,000	Plt 150,000	Plt 123,000	
BUN	60	59	45	43
Cr	1.6	1.7	1.4	1.2
Liver function test	Elevated	Elevated	Elevated	Normalized
Virology screen	Negative	Negative	Negative	Negative
Wound culture result for bacteria	Negative	Positive	Negative	Negative
Wound culture for candida	Negative	Negative‐ became positive at day 12 of admission	Positive‐ became negative at day 20 of admission	Negative
Blood culture for candida	Negative	Negative	Positive	Negative
Random blood sugar	103	120	140	100
BcHCO_3_−	19	24	28	Normal
Na	139	130	135	Normal
K	3.5	4	4.1	Normal

**FIGURE 1:**
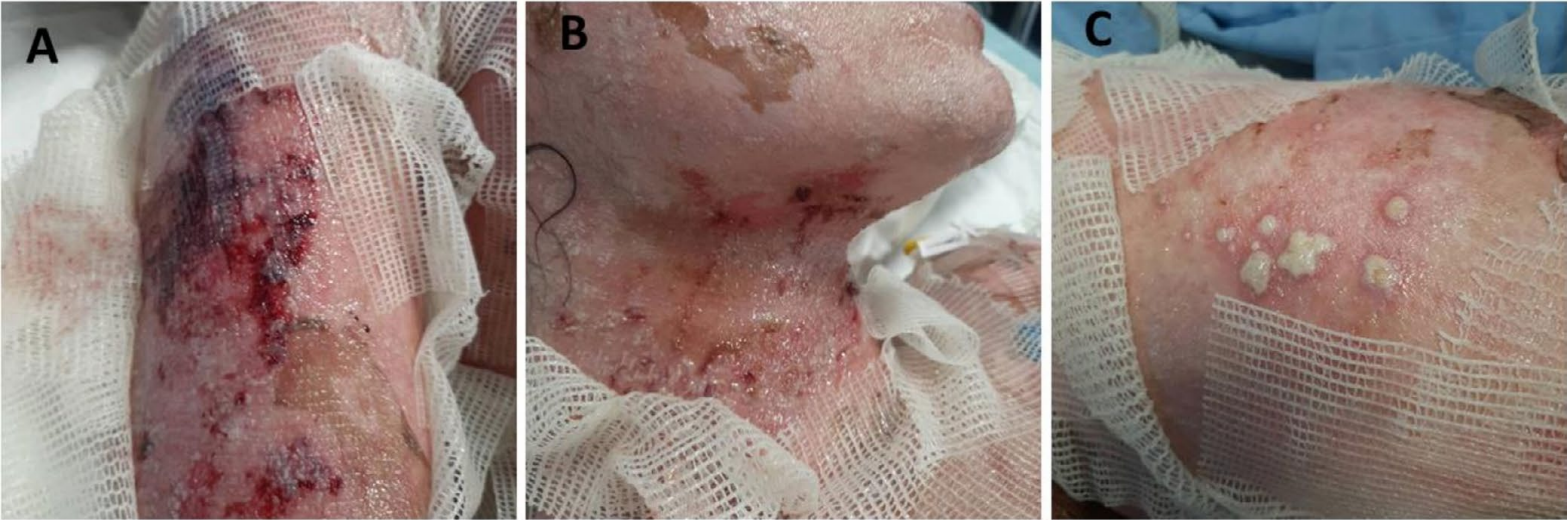
On Day 12 of admission, multiple satellite pustular lesions on the upper limb (A), neck and face (B), and leg (C) coincide with the presence of a systemic candidal infection.

The uneventful period after the IVIG injection was followed by pustular lesions and hemorrhagic crusts on the 12th day of admission, which were associated with moderate fever. The new lesions developed at sites of previous erosions, including the upper trunk, extremities, and head (Figure 1A–C).

Smear from newly formed lesions and pustules revealed candidal infection. A dose of fluconazole 800 mg/Po/STAT and then 400/PO/daily was instituted without waiting for culture and blood results. The positive wound smear result prompted wound culture and blood culture for candidal infection, both of which were positive for candidal infection. By continuing antifungal medications in the next 5 days, pustular lesions reduced and overall clinical condition improved. On the 23rd day of her admission, the erosions were completely dry, with no pustular lesions or hemorrhagic crusts, stable vital signs, and signs of skin re‐epithelialization. After consultation with her nephrologist, the patient was discharged on 400 mg/daily fluconazole and low‐dose prednisolone for her IgA nephropathy.

### 2.2 Outcome and Follow‐Up

The causative organism was identified as *Candida albicans*; the progression timeline strongly suggests true candidemia: the initial skin smear was positive on Day 12, followed by the finalization of the first positive blood culture on Day 17 (from a sample drawn on Day 12). Subsequent blood cultures from Day 14 and Day 15 were also positive. Treatment success was demonstrated by three consecutive negative blood cultures taken on Days 17, 18, and 19, with final negative results reported on Days 21, 22, and 23, respectively.

Fluconazole was discontinued 2 weeks after discharge, providing a constant negative blood culture result and a lack of clinical manifestations of candidiasis. Deflazacort was marked as a red flag drug for her, and instructions were given to avoid this drug, while losartan and atorvastatin were reinstituted into the daily regimen.

## 3 Discussion

The patient in our study had a history of IgA nephropathy, an autoimmune disease that causes antibodies to build up in the kidneys. This buildup leads to glomerulonephritis and kidney damage. It is well established that damaged kidneys have a reduced ability to clear drugs, which can result in drug accumulation and potential adverse drug reactions [8].

Deflazacort, a systemic corticosteroid used to treat IgA nephropathy, is primarily eliminated by the kidneys. This mechanism, combined with the impaired kidney function associated with IgA nephropathy, may have contributed to the development of TEN in our patient and in previously reported cases of TEN in patients with IgA nephropathy who received Deflazacort [2]. A possible explanation for the co‐occurrence of IgA nephropathy and TEN is a shared genetic predisposition. In the future, IgA nephropathy might be considered a risk factor for TEN, alongside immunosuppressed individuals, slow acetylators, and specific HLA alleles.

While the patient was also taking losartan and atorvastatin, both of which have been linked to TEN, the only recent addition to their regimen was deflazacort [9, 10].

The late discontinuation of deflazacort, 1 week after the skin eruption, may have hindered the resolution of the lesions and increased the risk of complications. Despite the delayed discontinuation of the culprit drug, early initiation of combination therapy with systemic corticosteroids, IVIG, and infliximab effectively halted disease progression and reduced the SCORTEN score within 6 days of admission. This combination therapy appears to be superior to systemic corticosteroid monotherapy [11]. Cyclosporine was not a suitable option due to its nephrotoxic potential and risk of exacerbating kidney damage.

The patient's course was complicated by both fungal and bacterial septicemia, a serious condition associated with high mortality rates in TEN patients. The development of septicemia, characterized by persistent fever, hypotension, asthenia, and positive blood and wound cultures, warranted the addition of a TNF inhibitor as an adjunctive therapy. Previous studies have demonstrated the benefit of TNF inhibitors in TEN patients with fungal or bacterial septicemia [12]. The patient's sepsis was treated with fluconazole, meropenem, and colistin. While fluconazole and meropenem have been associated with TEN [7, 13], colistin appears to be safe in this context [14]. None of the added medications worsened the skin lesions.

Fluconazole at a dose of 10 mg/kg per day appears to be a safe and effective antifungal drug in the treatment of candidial fungemia; nevertheless, the liver enzymes should be monitored to exclude raised liver enzymes, and therapy should be continued until negative blood culture and resolution of clinical manifestations [15].

In our study, wound culture for Candida was positive after nearly 12 days from admission; then blood fungemia was documented 5 days after positive wound culture. The usual source of blood fungemia in patients with TEN is the skin; we suggest the same source in our case [6]. Patients with TEN and fungal septicemia tend to be hospitalized for a longer duration [6]. Our patient was hospitalized for 23 days, which is close to the average median hospitalization in patients with TEN without sepsis [16].

## 4 Conclusion

Deflazacort could trigger Toxic Epidermal Necrolysis in patients with IgA nephropathy. In rare cases, invasive candidiasis may complicate the course of TEN, but early diagnosis through the visible clinical finding of diffuse pustules and hemorrhagic crusts and the early institution of antifungal medications could prevent further spread of the fungi to internal organs and subsequently early control of the fungal septicemia with a favorable outcome.

## Author Contributions

**Saman Al‐Zahawi:** writing – original draft, writing – review and editing. **Fatemeh Saberi:** writing – review and editing. **Tahereh Soori:** conceptualization. **Elham Yousefi:** conceptualization, supervision. **Kamran Balighi:** conceptualization, supervision, writing – original draft, writing – review and editing.

## Consent

Written informed consent for publication (including images) was obtained, IRB approval or a waiver was secured, as ethical approval was not required for this case report, in accordance with local guidelines.

## Conflicts of Interest

The authors declare no conflicts of interest.

## Data Availability

The data that support the findings of this study are not publicly available due to privacy reasons, but are available from the corresponding author upon request.
